# ﻿A new species of *Diglyphus* Walker (Hymenoptera, Eulophidae) from China, with notes on its biology and a key to the Chinese species

**DOI:** 10.3897/zookeys.1257.159532

**Published:** 2025-10-31

**Authors:** Wei-Jie Wan, Christer Hansson, Jian-Yang Guo, Wan-Xue Liu

**Affiliations:** 1 State Key Laboratory for Biology of Plant Diseases and Insect Pests, Key Laboratory for Prevention and Control of Invasive Alien Species of Ministry of Agriculture and Rural Affairs, Institute of Plant Protection, Chinese Academy of Agricultural Sciences, Beijing 100193, China Institute of Plant Protection, Chinese Academy of Agricultural Sciences Beijing China; 2 Biological Museum (Entomology), Lund University, Sölvegatan 37, SE-22362 Lund, Sweden Lund University Lund Sweden; 3 Natural History Museum, Life Sciences, Cromwell Road, London, UK Natural History Museum London United Kingdom

**Keywords:** Agromyzidae, biocontrol, parasitoid wasp, *Phytomyza vitalbae*, taxonomy

## Abstract

A new species of *Diglyphus* Walker, *D. albifemur* Liu, Hansson & Guo, sp. nov., is described based on extensive material from China collected from 2016 to 2024. The entire material included here has been reared from agromyzid leafminers (Diptera, Agromyzidae), mainly from *Phytomyza vitalbae* Kaltenbach, mining leaves of *Clematis orientalis* L. The new species is morphologically similar to the European species *D. clematidis* Navone & Hansson and shares the same host plant genus (*Clematis*) but can be distinguished by several diagnostic features detailed herein. Mitochondrial cytochrome c oxidase subunit I (COI) sequences of the new species are provided. The identification key to Chinese *Diglyphus* species by Zhu et al. (2000) was extended to incorporate the new species and all other Chinese species described since that publication.

## ﻿Introduction

The genus *Diglyphus* (Hymenoptera, Eulophidae) was described by Walker (1844). Since the original description several authors have contributed to the knowledge of the genus. Species descriptions from various parts of the world include Walker (1838, 1844), England; Girault (1916), North America; Erdős (1958), Hungary; Khan (1985), Arifa and Khan (1992), and Ahmad et al. (2013) (all from India); Ubaidillah and Yefremova (2001), Kazakhstan; Yefremova (2007), Yemen; Hesami et al. (2008), Iran; Yefremova et al. (2011), Turkey; and Wan et al. (2023), China. More extensive reviews of *Diglyphus* include Gordh and Hendrickson (1979) (worldwide fauna), Zhu et al. (2000) (Chinese fauna), and Hansson and Navone (2017) (European fauna).

The genus currently includes 42 species and is recorded in 67 countries (UCD Community 2023). Furthermore, 18 species of the genus are recorded from China (Zhu et al. 2000; Ye et al. 2018; Noyes 2019; Wan et al. 2023). The genus attacks Diptera (Agromyzidae, Ephydridae) and Lepidoptera (Gelechiidae, Gracillariidae, Lyonetiidae, Nepticulidae, Tortricidae) pests (UCD Community 2023), and thus it has economic significance.

Here we describe a new species of *Diglyphus* based on an extensive series of specimens collected across 31 provinces of China between 2016 and 2024. In addition to traditional morphological characterization, we provide COI sequences of the new species. Also, we extended the identification key to Chinese *Diglyphus* species (Zhu et al. 2000) to incorporate the new species, all other Chinese species described since 2000, and diagnostic characters for both sexes, where possible.

## ﻿Materials and methods

### ﻿Sampling

We collected leaves, particularly those of vegetables and ornamental plants, infested with agromyzid leafminers in China from 2016 to 2024. The leaves had been placed in cages, and each cage was maintained in climate chambers set at 25 ± 1 °C, 30–50% relative humidity, and a photoperiod of 14:10 h (light: dark) until agromyzid leafminers or parasitoids emerged. The specimens were preserved in anhydrous ethanol and maintained at –20 °C. The holotype of the new *Diglyphus* species is deposited in Insect Collection, the Institute of Zoology (**IOZ**), Chinese Academy of Sciences (**CAS**), Beijing, China. The paratypes and additional material are deposited in the Institute of Plant Protection (**IPP**), Chinese Academy of Agricultural Sciences (**CAAS**), Beijing, China.

### ﻿Morphological identification and documenting methods

The specimens were examined using a stereomicroscope (Olympus, SZX-16). Photographs were taken using an Olympus BX43 microscope equipped with a Helicon Focus system.

The morphological terminology and measurement methods follow Gibson (1989), Gauthier et al. (2000), Hansson (1990), and Yefremova et al. (2011), with the following abbreviations being used in the text:

**F1–2** maximum length of flagellomeres 1–2.

**POL** Posterior ocellar line: shortest distance between the inner margins of the lateral ocelli.

**OOL** Ocular ocellar line: shortest distance between the inner margin of a lateral ocellus and the inner margin of the compound eye.

### ﻿Molecular methods

Six specimen (3 female, 3 male) were reared from *Phytomyza vitalbae* on leaves of *Clematis orientalis* on 4 June 2023 in Gansu Province, China. Genomic DNA was extracted from the metasoma of each specimen. The extraction methods followed those described by De Barro and Driver (1997), with some modifications. DNA extraction was performed using a 200 µL microcentrifuge tube (Bioevopeak, Shandong, China) and 200-µL pipette tip (Bioevopeak) sealed by heating to grind the metasoma into a homogenate. The homogenate was incubated at 65 °C, 25 °C, and 96 °C for 30 min, 2 min, and 10 min, respectively.

After extraction, the genomic DNA was stored at −20 °C until molecular diagnosis. The COI gene (625 bp) was amplified using the primers COI1490 and HCO2198 (Folmer et al. 1994). Amplifications were performed as described by Hebert et al. (2003) and Wan et al. (2023). The unpurified PCR products were sent to Sangon Biotech Co., Ltd, Beijing, China, for bidirectional sequencing, and primers were designed by Sangon Biotech Co., Ltd, Beijing, China.

## ﻿Results

### ﻿Key to the Chinese species of *Diglyphus*

The following key is modified from Zhu et al. (2000), with some couplets and diagnostic characters adapted from Hansson and Navone (2017). The quotation marks are used exclusively to denote direct quotations from the two references. It has been updated to include the new species described here and four additional species now known from China: *D. bulbus*, *D. difasciatus*, *D. intermedius*, and *D. wani*.

**Table:** 

1	Basal part of cubital vein curved forwards towards anterior margin of wing; setae at base of wing short and uniform, dense; speculum absent, or if present then small and present only along anterior margin of wing; “wings long and narrow” (fore wing 1.76–3.10× as long as wide) (Fig. 4C, F)	**2**
–	Basal part of cubital vein not distinctly curved forwards at base, setae not so short and uniform; “speculum generally present and extending posteriorly to cubital vein” (although sometimes very small or absent) (Fig. 4A, C)	**4**
2	Scape and tibiae white; speculum small, “present just posterior to parastigma”; “known from male only”	***D. albitibiae* Zhu, LaSalle & Huang**
–	“Scape dark with metallic shine”; tibiae pale with dark markings; “speculum completely absent”	**3**
3	“Tibiae metallic with apex of hind tibiae yellow; setae on wings dark”	**D. metallicus Zhu, LaSalle & Huang**
–	“Tibiae metallic with base and apical 1/6–1/5 yellow”; setae on wings pale	***D. isaea* Walker**
4	Fore wings partly infuscate (Fig. 4B)	**5**
–	Fore wings completely hyaline (Fig. 4A, C, F)	**6**
5	Fore wings with an infuscate spot below parastigma and below stigmal vein	***D. bimaculatus* Zhu, LaSalle & Huang**
–	Fore wings with complete vertical infuscate bands below base of marginal and stigmal veins	***D. difasciatus* Liu, Hansson & Wan**
6	Scape completely pale, or dark with basal 1/5–2/3 pale	**7**
–	Scape completely dark	**12**
7	Male scape swollen (scape 1.36× as long as broad) (Fig. 4D)	***D. bulbus* Ubaidillah & Yefremova**
–	Male scape slender (scape more than 3.10× as long as broad)	**8**
8	Wing veins white	***D. albinervis* Zhu, LaSalle & Huang**
–	Wing veins yellowish to brown	**9**
9	Notauli complete (Fig. 4F)	***D. pustenzis* Erdös & Novicky**
–	Notauli incomplete	**10**
10	Hind femora white with base infuscate, occasionally male hind femora with basal 1/2 dark brown	***D. albifemur* sp. nov.**
–	Hind femora with basal 1/3–3/4 dark	**11**
11	Male gaster with a pale spot (Fig. 4E)	***D. albiscapus* Erdös**
–	Male gaster completely dark	***D. pulchripes* (Ashmead)**
12	Male wings with enlarged veins	**13**
–	Male wings with slender veins (as in female)	**15**
13	Speculum very small, or absent	***D. inflatus* Zhu, LaSalle & Huang**
–	Speculum present and relatively large	**14**
14	“Fore and middle tibiae yellow”	***D. pachyneurus* Graham**
–	All tibiae dark with apical 1/4 pale	***D. crassinervis* Erdös**
15	Fore tibiae pale	***D. begini* (Ashmead)**
–	Fore tibiae mainly dark	**16**
16	Mid and hind tibiae with base pale	**17**
–	Mid and hind tibiae 3/4 dark	**18**
17	Mid and hind tibiae 1/5–1/4 metallic green	***D. gibbus* Zhu, LaSalle & Huang**
–	Mid and hind tibiae 1/4–1/3 black	***D. intermedius* (Girault)**
18	“Posterior setae on midlobe of mesoscutum reach the transscutal articulation”	***D. minoeus* (Walker)**
–	Posterior setae on midlobe of mesoscutum do not reach the transscutal articulation	***Diglyphus wani* Liu, Zhu & Yefremova**

#### ﻿ Diglyphus albifemur

Taxon classificationAnimaliaHymenopteraEulophidae

Liu, Hansson & Guo sp. nov.

CB3B0E65-BE6D-5390-836F-2F9F61252DF1

https://zoobank.org/1360515B-2073-40B6-8EC5-93F39B898C0A

Figs 1, 2, 3

##### Material.

***Holotype***: • ♀ China, Gansu Province, Jiuquan City; 40°10'34"N, 94°43'56"E; 4 June 2023; Bao-Wei Huo leg.; reared from *Phytomyza vitalbae* on leaves of *Clematis orientalis*, deposited in CAS (accession number, IOZ(E)224808). ***Paratypes*** (deposited in IPP): • 3♀ 1♂ with same label data as holotype (accession numbers, IPP(Da)PL1–IPP(Da)PL4); • 1♀ China, Shandong Province, Yantai City, 37°17'26"N, 121°33'46"E, 21 May 2017, Qin-Min Yang leg., reared from leaves of *Sonchus oleraceus* (accession numbers, IPP(Da)MP1); • 5♀ China, Xinjiang Uygur Autonomous Region, Kashi City, 39°19'25"N, 76°01'11"E, 25 July 2017, Jun Zhong leg., reared from leaves of *Clematis orientalis* (accession numbers, IPP(Da)SL1–IPP(Da)SL5); • 1♀ China, Gansu Province, Jiuquan City, 40°07'04"N, 94°37'55"E, 18 June 2018, Su-Jie Du leg., reared from leaves of *Pisum sativum* (accession numbers, IPP(Da)QL1); • 1♂ China, Heilongjiang Province, Jiagedaqi District, 50°25'09"N, 91°46'29"E, 6 July 2018, Xin-Fei Cheng leg., reared from *Phytomyza horticola* on leaves of *Pisum sativum* (accession numbers, IPP(Da)GG1); • 3♀ 3♂ China, Xizang, Shannan City, 29°14'21"N, 91°46'28"E, 4 August 2018, Yu-Jun Zhong leg., reared from *Phytomyza horticola* and *Liriomyza huidobrensis* on leaves of *Lepidium latifolium* (accession numbers, IPP(Da)SN1–IPP(Da)SN6); • 1♀ China, Neimenggu Autonomous Region, Bayannur, 40°45'55"N, 107°24'07"E, 7 September 2018, Su-Jie Du leg., reared from *Phytomyza horticola* on leaves of *Solanum nigrum* (accession numbers, IPP(Da)LH1).

##### Additional material (deposited in IPP).

26♀ 16♂ China, Xinjiang Uygur Autonomous Region, Kizilsu Kirgiz Autonomous Prefecture, 39°56'53"N, 75°33'28"E, 30 June 2018, Xiao-Qing Xian leg., reared from leaves of *Raphanus sativus* (accession numbers, IPP(Da)KZ1–IPP(Da)KZ42); • 4♀ China, Neimenggu Autonomous Region, Alxa League, 41°56'32"N, 101°03'32"E, 8 September 2018, Su-Jie Du leg., reared from *Phytomyza vitalbae* on leaves of *Vigna unguiculata* (accession numbers, IPP(Da)EJ1–IPP(Da)EJ4); • 10♀ 1♂ China, Neimenggu Autonomous Region, Alxa League, 41°56'32"N, 101°03'32"E, 8 September 2018, Su-Jie Du leg., reared from *Phytomyza vitalbae* on leaves of *Raphanus sativus* (accession numbers, IPP(Da)AL1–IPP(Da)AL11); • 2♀ China, Gansu Province, Jiuquan City, 40°09'54"N, 94°41'14"E, 25 September 2022, Su-Jie Du leg., reared from *Phytomyza vitalbae* on leaves of *Clematis orientalis* (accession numbers, IPP(Da)ZH1–IPP(Da)ZH2); • 15♀ 33♂ China, Gansu Province, Jiuquan City, 40°15'11"N, 94°45'22"E, 2 June 2023, Bao-Wei Huo leg., reared from *Phytomyza vitalbae* on leaves of *Clematis orientalis* (accession numbers, IPP(Da)DH1–IPP(Da)DH48); • 10♀ 17♂ China, Gansu Province, Jiuquan City, 35°18'48"N, 107°02'35"E, 3 June 2023, Bao-Wei Huo leg., reared from *Phytomyza vitalbae* on leaves of *Clematis orientalis* (accession numbers, IPP(Da)CX1–IPP(Da)CX17); • 16♀ 16♂ China, Gansu Province, Jiuquan City, 40°10'03"N, 94°42'52"E, 4 June 2023, Bao-Wei Huo leg., reared from *Phytomyza vitalbae* on leaves of *Clematis orientalis* (accession numbers, IPP(Da)JQ1–IPP(Da)JQ32); • 2♀ 8♂ China, Gansu Province, Jiuquan City, 40°09'59"N, 94°42'31"E, 4 June 2023, Bao-Wei Huo leg., reared from *Phytomyza vitalbae* on leaves of *Clematis orientalis* (accession numbers, IPP(Da)HZ1–IPP(Da)HZ10); • 121♀ 224♂ China, Gansu Province, Jiuquan City, 40°09'59"N, 94°43'17"E, 4 June 2023, Bao-Wei Huo leg., reared from *Phytomyza vitalbae* on leaves of *Clematis orientalis* (accession numbers, IPP(Da)QS1–IPP(Da)QS345); • 24♀ 30♂ with same label data as holotype; • 13♀ 25♂ China, Gansu Province, Jiuquan City, 40°09'46"N, 94°43'19"E, 4 June 2023, Bao-Wei Huo leg., reared from *Phytomyza vitalbae* on leaves of *Clematis orientalis* (accession numbers, IPP(Da)AD1–IPP(Da)AD38).

##### Diagnosis.

**Figure 1.**
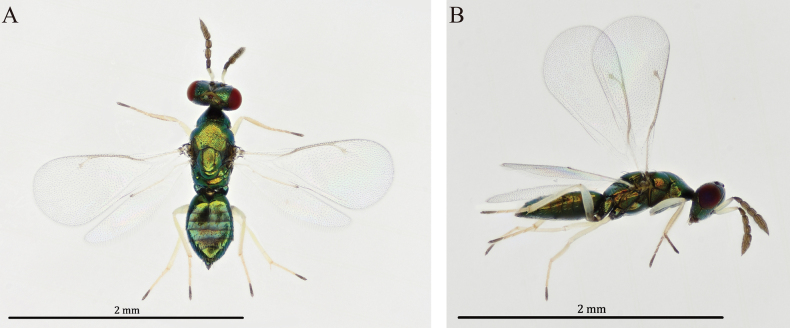
*Diglyphus albifemur* sp. nov. female, holotype, habitus. **A.** Dorsal view; **B.** Dorsal view.

Scape entirely white, with only the apex slightly infuscate (Fig. 3B). With yellow marking on the ocellus (Fig. 3A). Mesosoma and metasoma metallic green with golden luster (Figs 1, 3A). Fore wing speculum bare; postmarginal vein 1.55× as long as stigmal vein in the female, and 1.50× in the male. All femora white, except for the narrowly infuscate base of the hind femur in the female, occasionally dark brown in basal ½ in the male; all tibiae white.

##### Description.

**Female** (Fig. 1). Body length 1.54 mm. Fore wing length 1.07 mm. Scape white with apex infuscate. Pedicel dark brown. Flagellum brown, with antennal sensilla yellowish. Head dark brown with golden-green luster, and with yellow markings on the ocellus. Eyes red and ocelli brown. Mandibles pale brown. Pronotum, mesoscutum, scutellum, dorsellum, and propodeum metallic green. Coxae dark brown with golden-green reflections; femora white, hind femora with base infuscate; tibiae white; fore tarsi dusky, mid and hind tarsi with tarsomeres 1–3 white, 4 dark brown. Wings hyaline, veins yellowish. Female gaster metallic green with golden or bronze luster, but male gaster dark brown with golden-green reflections.

***Head*** (Fig. 3A, C). Head length 0.50× width in dorsal view (Fig. 3A). Antennal flagellum with two funiculars and three clavomeres; scape 4.17× as long as broad and 2.50× as long as pedicel; pedicel 1.67× as long as broad; F1 1.18× and F2 1.10× as long as broad, F1 1.68× as long as F2; clava 2.83× as long as broad, 1.09× as long as scape (Fig. 3B). Antennal toruli situated above the level of lower margin of eyes. POL 1.86× OOL. Malar sulcus present; malar space 0.47× eye height (Fig. 3C). Frons and vertex with distinct reticulation. Eyes with sparse and short setae. Maxillary palpi with two segments.

***Mesosoma*** (Fig. 3A). Pronotum 0.46× as long as mesoscutum. Mesoscutum 1.14× as long as scutellum; mid lobe with two pairs of long setae; notauli incomplete. Scutellum as long as broad, with straight sublateral grooves and two pairs of setae. Setae on mesoscutum and scutellum dark brown. Dorsellum with reticulation. Propodeum obviously shorter than scutellum and without median carina; propodeal callus with three setae.

***Wings*** (Fig. 3E). Fore wing 1.76× as long as wide; with seven setae on dorsal surface of submarginal vein. Speculum almost bare, with a few scattered setae. Costal cell with two rows of setae, including 16 setae at the base and an incomplete row with 14 setae in apical part. Postmarginal vein 1.55× as long as stigmal vein. Cubital vein straight at base.

***Metasoma*** (Fig. 3D). Petiole inconspicuous. Gaster short ovate, 1.57× as long as wide in dorsal view; apex acute. Tip of ovipositor sheaths visible in dorsal view.

**Figure 2.**
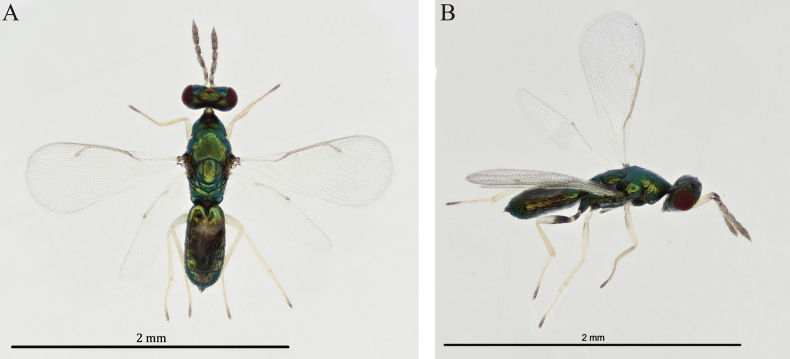
*Diglyphus albifemur* sp. nov. male, paratype, habitus. **A.** Dorsal view; **B.** Dorsal view.

**Male** (Fig. 2). Similar to the female, but generally smaller. Body length 1.5 mm. Hind femora completely white to occasionally with basal ½ dark brown. Gastral tergites mainly black, tergites 1 and 7 with golden-green reflections. Head 0.56× as long as wide in dorsal view. POL 2.40× OOL. Scape 4.0× as long as broad, 2.0× as long as pedicel. Pedicel 2.50× as long as broad. F1 2.20× and F2 1.46× as long as broad, F1 1.38× as long as F2. Clava 3.0× as long as broad, 2.05× as long as scape and 2.81× as long as F2. Fore femora with basal ½ dark brown. Mesoscutum 1.67× as long as scutellum. Scutellum as long as broad. Fore wing 1.80× as long as wide. Postmarginal vein 1.50× as long as stigmal vein. Gaster 2.03× as long as wide in dorsal view.

**Figure 3.**
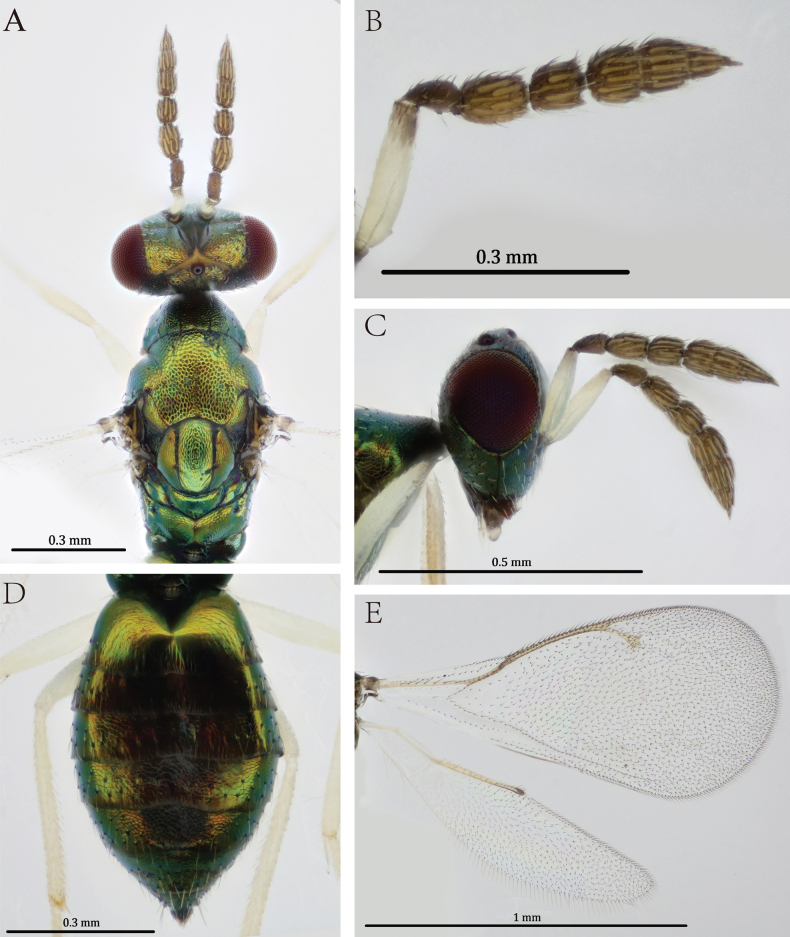
*Diglyphus albifemur* sp. nov. **A.** Female paratype, head and mesosoma, dorsal view; **B.** Female paratype, antenna, lateral view; **C.** Female paratype, head, lateral view; **D.** Female paratype, metasoma, dorsal view; **E.** Female paratype, right fore and hind wings.

##### Hosts and biology.

The new species was reared mainly from *Phytomyza vitalbae* Kaltenbach on leaves of *Clematis orientalis* L.; a few specimens were reared from *Phytomyza horticola* Goureau on leaves of *Vigna unguiculata* (L.) Walp. and *Raphanus sativus* L. Other Eulophidae species that were reared from the above leaf-miners include: *Neochrysocharis formosa* (Westwood), *Diglyphus isaea* (Walker), *D. wani* Liu, Zhu & Yefremova, *D. pusztensis* (Erdös & Novicky), and *Closterocerus lyonetiae* (Ferrière).

**Figure 4.**
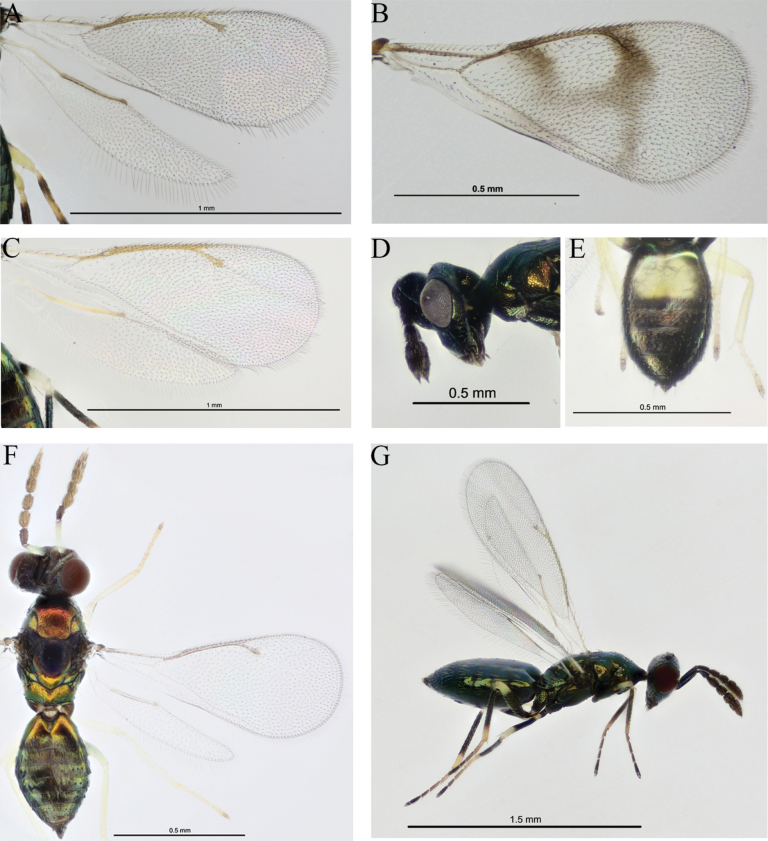
Six *Diglyphus* species. **A.***D. isaea*, wings; **B.***D. difasciatus*, fore wing; **C.***D. crassinervis*, wings; **D.***D. bulbus*, head, lateral view; **E.***D. albiscapus*, metasoma, dorsal view; **F.***D. pusztensis*, dorsal view; **G.***D. isaea*, habitus, lateral view.

##### Distribution.

China (Gansu, Heilongjiang, Neimenggu, Shandong, Xizang, and Xinjiang).

##### Etymology.

The species name is derived from a combination of the Latin *albus* (white) and *femur*, referring to the main colour of femora.

##### Comments.

*Diglyphus albifemur* is similar to *D. clematidis* Navone & Hansson, but *D. albifemur* differs from *D. clematidis* in having: (1) mesoscutum and scutellum golden-green (purple, or golden-green with purplish reflections in *D. clematidis*); (2) postmarginal vein 1.50–1.55× as long as stigmal vein (postmarginal vein 1.1× as long as stigmal vein in *D. clematidis*); (3) wing veins in male not enlarged (male marginal vein distinctly enlarged in *D. clematidis*); (4) tergites of male gaster mainly black, tergites 1, 6, and 7 with golden-green reflections (male gaster with a large pale spot subbasally in *D. clematidis*).

##### Molecular analysis.

Five haplotypes were detected from six specimen of *Diglyphus albifemur* sp. nov., based on COI. The COI sequence of *D. albifemur* sp. nov. was uploaded to GenBank (GenBank accession numbers PV920650–PV920655).

## Supplementary Material

XML Treatment for Diglyphus albifemur

## ﻿Additional information

### Use of AI

No use of AI was reported.

### Author ORCIDs

Wei-Jie Wan https://orcid.org/0009-0007-2972-3009

Christer Hansson https://orcid.org/0000-0002-9429-0197

### Data availability

All of the data that support the findings of this study are available in the main text.
